# From bit to it: How a complex metabolic network transforms information into living matter

**DOI:** 10.1186/1752-0509-1-33

**Published:** 2007-07-30

**Authors:** Andreas Wagner

**Affiliations:** 1 Department of Biochemistry, University of Zurich, Building Y27, Winterthurerstrasse 190, CH-8057 Zurich, Switzerland

## Abstract

**Background:**

Organisms live and die by the amount of information they acquire about their environment. The systems analysis of complex metabolic networks allows us to ask how such information translates into fitness. A metabolic network transforms nutrients into biomass. The better it uses information on available nutrient availability, the faster it will allow a cell to divide.

**Results:**

I here use metabolic flux balance analysis to show that the accuracy I (in bits) with which a yeast cell can sense a limiting nutrient's availability relates logarithmically to fitness as indicated by biomass yield and cell division rate. For microbes like yeast, natural selection can resolve fitness differences of genetic variants smaller than 10-6, meaning that cells would need to estimate nutrient concentrations to very high accuracy (greater than 22 bits) to ensure optimal growth. I argue that such accuracies are not achievable in practice. Natural selection may thus face fundamental limitations in maximizing the information processing capacity of cells.

**Conclusion:**

The analysis of metabolic networks opens a door to understanding cellular biology from a quantitative, information-theoretic perspective.

## Background

Organisms need to acquire information about their environment in order to survive and reproduce. They need to respond to information about changes in temperature, soil conditions, water availability, nutrient supply, predation pressure, and many other factors. The ability to acquire and use such information arguably affects organismal fitness [1-6]. However, we know nothing about the quantitative relationship between such information and fitness.

In microbes, an important fitness component is a cell's growth or cell division rate. The selection pressure to grow rapidly during times of nutrient availability has left clear traces in the evolutionary record, such as strong microbial codon usage biases that allow high translation efficiency of abundant proteins [7,8]. It has recently become possible to make quantitative predictions about a cell's maximal division rate, based on nearly complete information about the metabolic networks that sustain cellular life [9-16]. These metabolic networks comprise of the order of 10^3 ^chemical reactions for free-living organisms. Their structure has been elucidated in several organisms by manual curation, aided by functional genomic data [9,11,12]. Flux balance analysis allows one to predict those flows of matter – metabolic fluxes – through each reaction of a network that are consistent with the laws of mass conservation. More precisely, flux balance analysis predicts ratios of metabolic fluxes, i.e., values of metabolic fluxes relative to a reference flux that needs to be determined independently, for example through experimental measurements. Together with the known biomass composition of an organism, flux balance analysis can then also identify the metabolic fluxes that maximize biomass production. For organisms such as *Saccharomyces cerevisiae *and common growth substrate compositions, such as minimal media with glucose as a sole carbon source, the predictions of flux balance about maximal biomass yield are in good agreement with experimental data, where available [14,17,18]. For other organisms and more unusual environments [19-21] this does not always hold. Strikingly, however, even in this case laboratory evolution experiments can produce strains of organisms that show the maximally predicted biomass yield within a short amount of time [13,19,20,22]. I here use flux balance analysis of the yeast metabolic network to explore the relationship between environmental information and how rapidly an organism produces biomass per unit time.

The kind and concentration of available growth substrates influence a cell's maximal biomass yield [23-26]. Cells have developed elaborate nutrient sensing mechanisms to respond to changes in nutrient abundances. For example, in yeast, dedicated glucose sensor proteins (*Snf*3, *Rgt*2) as well as glycolytic intermediates form the beginning of a signaling cascade. This cascade produces an integrated cellular response that includes the expression of glucose transporter genes (*HXT1*-*HXT7*), the expression of glycolytic genes, as well as the repression of many other genes [24,25]. Even though long-studied, glucose sensing is still only qualitatively and incompletely understood. This holds to an even greater extent for other sensing mechanisms, such as those for phosphate and nitrogen [24,25]. The accuracy of the sensing mechanism is clearly important for optimal growth, but there is an important asymmetry: Overestimation of a nutrient concentration, e.g. through overexpression of catabolic genes, will not lead to sub-optimal growth due to metabolic undercapacity, because the available nutrients can still be maximally used. In contrast, underestimation and the resulting undercapacity will lead to reduced growth. Information is thus of greatest value from a metabolic perspective if it prevents underestimation of nutrient concentrations, and thus undercapacity of a nutrient utilization system. I will thus focus primarily on the consequences of sensing errors that lead to underestimates of nutrient concentrations. Note that overestimation of nutrient concentration may lead to sub-optimal growth for other reasons, reasons that cannot currently be modeled in a metabolic context, and that are discussed below.

Information acquired through nutrient sensing can be represented as follows. Consider *k *nutrients and their actual concentrations *N*_*i *_(1 ≤ *i *≤ *k*) in a cell's environment. If a cell underestimates the actual nutrient concentration in the environment for any one nutrient, then its "measurement" Nim
 MathType@MTEF@5@5@+=feaafiart1ev1aaatCvAUfKttLearuWrP9MDH5MBPbIqV92AaeXatLxBI9gBaebbnrfifHhDYfgasaacH8akY=wiFfYdH8Gipec8Eeeu0xXdbba9frFj0=OqFfea0dXdd9vqai=hGuQ8kuc9pgc9s8qqaq=dirpe0xb9q8qiLsFr0=vr0=vr0dc8meaabaqaciaacaGaaeqabaqabeGadaaakeaacqWGobGtdaqhaaWcbaGaemyAaKgabaGaemyBa0gaaaaa@30BC@ of the actual nutrient concentration is such that Nim
 MathType@MTEF@5@5@+=feaafiart1ev1aaatCvAUfKttLearuWrP9MDH5MBPbIqV92AaeXatLxBI9gBaebbnrfifHhDYfgasaacH8akY=wiFfYdH8Gipec8Eeeu0xXdbba9frFj0=OqFfea0dXdd9vqai=hGuQ8kuc9pgc9s8qqaq=dirpe0xb9q8qiLsFr0=vr0=vr0dc8meaabaqaciaacaGaaeqabaqabeGadaaakeaacqWGobGtdaqhaaWcbaGaemyAaKgabaGaemyBa0gaaaaa@30BC@ <*N*_*i*_. Now subdivide the interval (0, *N*_*i*_) into *n*_*i *_equal subintervals. If the cell can place (measure) the concentration of *i *within the interval (*N*_i_(*n*_i_-1)/*n*_i_, *N*_i_) then it has *I*_*i *_= log_2 _*n*_*i *_bits of information about this nutrient. If the measurement error, when viewed as a random variable, has a symmetric or a uniform distribution *within this interval*, then the expected sensing error is *E*_*i *_= *N*_*i*_/2*n*_*i*_. Nutrient information and sensing error thus relate to each other as

Ii=log⁡2(Ni2Ei)
 MathType@MTEF@5@5@+=feaafiart1ev1aaatCvAUfKttLearuWrP9MDH5MBPbIqV92AaeXatLxBI9gBaebbnrfifHhDYfgasaacH8akY=wiFfYdH8Gipec8Eeeu0xXdbba9frFj0=OqFfea0dXdd9vqai=hGuQ8kuc9pgc9s8qqaq=dirpe0xb9q8qiLsFr0=vr0=vr0dc8meaabaqaciaacaGaaeqabaqabeGadaaakeaacqWGjbqsdaWgaaWcbaGaemyAaKgabeaakiabg2da9iGbcYgaSjabc+gaVjabcEgaNnaaBaaaleaacqaIYaGmaeqaaOWaaeWaaeaadaWcaaqaaiabd6eaonaaBaaaleaacqWGPbqAaeqaaaGcbaGaeGOmaiJaemyrau0aaSbaaSqaaiabdMgaPbqabaaaaaGccaGLOaGaayzkaaaaaa@3D89@

Although *E*_*i *_and *I*_*i *_are equivalent, using *I*_*i *_has two advantages. First, its units (bits) are canonical measures of information [27]; second, information content is additive, that is, if a cell has *I*_*j *_bits of information on nutrient *j *(1 ≤ *j *≤ *k*), then it has I=∑jIj
 MathType@MTEF@5@5@+=feaafiart1ev1aaatCvAUfKttLearuWrP9MDH5MBPbIqV92AaeXatLxBI9gBaebbnrfifHhDYfgasaacH8akY=wiFfYdH8Gipec8Eeeu0xXdbba9frFj0=OqFfea0dXdd9vqai=hGuQ8kuc9pgc9s8qqaq=dirpe0xb9q8qiLsFr0=vr0=vr0dc8meaabaqaciaacaGaaeqabaqabeGadaaakeaacqWGjbqscqGH9aqpdaaeqaqaaiabdMeajnaaBaaaleaacqWGQbGAaeqaaaqaaiabdQgaQbqab0GaeyyeIuoaaaa@34A6@ bits of information about its entire nutrient environment, if the nutrients occur independently from one another, or if the cell measures them independently from each other.

Flux balance analysis allows us to immediately assess the fitness value of nutrient information for a cell, because a cell's maximal biomass yield is a function of the measured nutrient concentrations (N1m,…,Nkm)
 MathType@MTEF@5@5@+=feaafiart1ev1aaatCvAUfKttLearuWrP9MDH5MBPbIqV92AaeXatLxBI9gBaebbnrfifHhDYfgasaacH8akY=wiFfYdH8Gipec8Eeeu0xXdbba9frFj0=OqFfea0dXdd9vqai=hGuQ8kuc9pgc9s8qqaq=dirpe0xb9q8qiLsFr0=vr0=vr0dc8meaabaqaciaacaGaaeqabaqabeGadaaakeaacqGGOaakcqWGobGtdaqhaaWcbaGaeGymaedabaGaemyBa0gaaOGaeiilaWIaeSOjGSKaeiilaWIaemOta40aa0baaSqaaiabdUgaRbqaaiabd2gaTbaakiabcMcaPaaa@390D@ and the extent to which these concentrations differ from their actual value (*N*_1 _,..., *N*_*k*_).

## Results

### Diminishing returns on improved information acquisition

**Figure 1:**
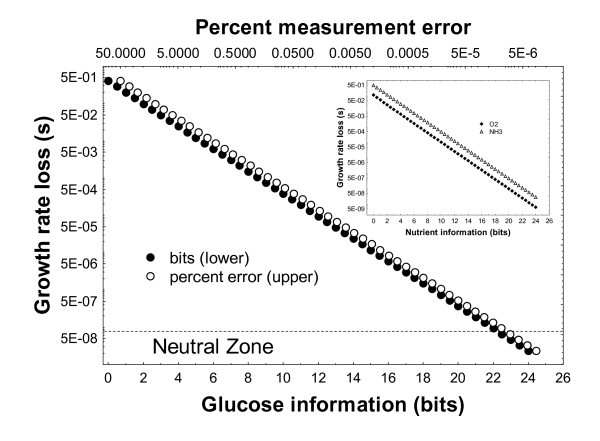
Increased glucose information (bits, lower horizontal axis, filled circles) and reduced sensing error (percent, upper horizontal axis, open symbols) cause an increases in fitness (1-*s*) as estimated through biomass production in the yeast metabolic network via flux balance analysis. The vertical axis shows the selection coefficient *s*, the difference to the maximal biomass yield at perfectly accurate information. Results are nearly identical for the other four substrates, and are shown for O_2 _and NH_3 _in the inset. The dashed horizontal line demarcates a neutral zone, below which (s < 7.33 × 10^-8^; see text) growth rate increases are selectively neutral.

The relationship between information and fitness is best explored for a defined environment, such as a minimal growth medium. The environment I use contains NH_3_, inorganic phosphate (*P*_*i*_), sulfate, and glucose as the sole carbon source. Oxygen is available as a terminal electron acceptor. For simplicity, I first focus on a scenario where information about all substrates except glucose is perfectly accurate. I assume that the biomass yield *Y *per unit time is linearly proportional to a cell's division rate *G*, a measure of fitness. In other words, *Y *= *cG*, *c *being some constant. I express the effect of incomplete information on biomass yield *Y *as *s *= 1-*Y*/*Y*_max _= 1-*G*/*G*_max_, where *Y*_max _and *G*_max _are the maximally achievable biomass yields and cell division rates, respectively, i.e., the yields and rates for perfectly accurate glucose information. The quantity *s *can also be thought of as a selection coefficient, as a measure by how far a cell's fitness *w *= 1-*s *= *G*/*G*_max _is reduced by incomplete information. Figure 1 shows how a cell's fitness depends on the amount of information the cell can acquire about substrate concentration. Specifically, the figure shows that the logarithm of fitness depends linearly on information in bits. The relationship of *s *and information is especially simple if a binary logarithm is used to scale *s*, i.e., -log_2_(*s*) = I_*GLC*_+1. This simple relationship emerges numerically from flux balance analysis, but it also has a straightforward intuitive explanation. If zero bits of information are available for a growth-limiting nutrient, then under the assumptions used here, the cell's "guess" about nutrient concentrations will be randomly distributed in the interval (0, *N*_*i*_), with an expected value of *N*_*i*_/2. At this expected value, the division rate of a cell will be half the maximal growth rate, such that *s *= 1/2. The above relationship between *s *and *I *then holds, because -log_2_(1/2) = 1 = *I*+1. If one bit of information is available (*I *= 1), then the cell's measurement will be randomly distributed in the interval (*N*_*i*_/2, *N*_*i*_), with an expected value of 3*N*_*i*_/4, leading to s = 1/4, and -log_2_(1/4) = 2 = *I*+1. The same line of reasoning applies to ever increasing values of *I*. The key assumption in this intuitive explanation is that if one nutrient is growth-limiting, then cell division rate depends linearly on the cell's ability to utilize this nutrient. This is not obvious *a priori*, because the nutrient's metabolic products may be fed into many different pathways that produce essential biomass components. The distribution of these products among different pathways, and the cell's final resulting division rate, might in principle depend on the concentration of the nutrient *and *on that of other nutrients. However, flux balance analysis shows that the dependency between nutrient concentration and biomass yield is quite simple and linear.

Nutrient sensing has much greater impact if the amount of information acquired by a cell is low (Figure 1). For instance, an increase in available glucose information from 1 bit (low-high) to 2 bits (four distinct concentration values) causes a 36% increase in growth rate, whereas an increase from 14 to 15 bit causes an increase of 0.0012 percent. For the purpose of comparison, the figure also shows the relationship between fitness reduction and sensing error in percent. Fitness reduction *s *decreases linearly with decreasing sensing error (note the double-logarithmic scale). Quantitatively very similar linear-log and linear relationships hold for the other four nutrients (data for oxygen and ammonium are shown in the inset for Figure 1). In sum, the logarithm of division rate scales linearly with nutrient information in bits, and increased information acquisition carries diminishing fitness returns.

### Even very small sensing errors cause adaptively significant growth-rate differences

How large must a growth rate difference *s *(due to imperfect nutrient sensing) be in order to matter to natural selection? The influence of genetic drift dominates over that of natural selection, if a reduction in growth rate is *s *< 1/4*N*_*e *_for diploid cells, where *N*_*e *_is the effective population size [28,29]. *N*_*e*_, in turn, can be estimated from the synonymous nucleotide diversity π in a population, and the per-generation mutation rate μ as *N*_*e *_= π/4μ. Thus, if a growth rate difference is smaller than *s *= μ/π in a population, then the associated growth rate difference is too small to be seen by natural selection. The rate of mutations per nucleotide and generation in *Saccharomyces cerevisiae *has been estimated at μ = 2.2 × 10^-10 ^[30]. In the closest wild relative of yeast synonymous π has been estimated as π = 0.003 [31]. The above parameters yield *s *= 7.33 × 10^-8 ^as a "critical" growth rate difference that can still be seen by natural selection. No data on *synonymous *nucleotide diversity are available for *S. cerevisiae *itself, but a recent estimate [32] on *overall *nucleotide diversity of π = 0.0046 (which is typically smaller than synonymous diversity) suggests an upper bound of *s *= 4.78 × 10^-8^, rendering the critical *s *I use here conservative. Growth rate differences below this value are more strongly influenced by drift than by selection.

The critical selection coefficient *s *is indicated in Figure 1 by a horizontal line. Below this line, any gains in information do have negligible effect. Specifically, information gains exceeding *I *= -log_2 _*s*-1 ≈ 22 bits are selectively neutral for yeast.

### Reduced fitness value of information for imperfect sensing of several nutrients

**Figure 2:**
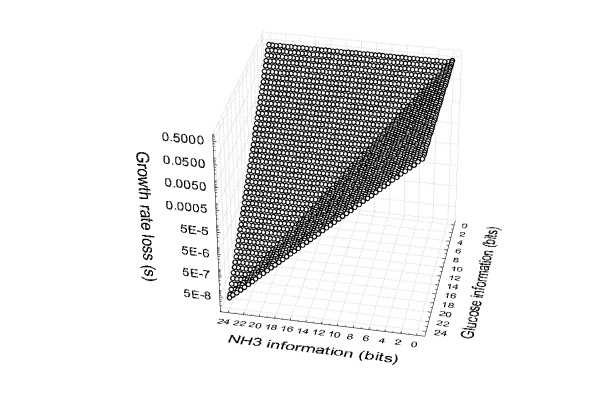
Dependency of the selection coefficient *s *on glucose information and ammonium information.

Thus far, I assumed limited information for only one nutrient, but what if information is limited for more than one nutrient? Consider a genotype that systematically underestimates the availability of one substrate, such as glucose, because of a poor sensing mechanism. The resulting undercapacity to metabolize this substrate renders the substrate growth-limiting. In this situation, accurate sensing of the availability of another substrate, such as ammonium, may not increase fitness. The reason is that growth is limited not by a lack of information about ammonium, but by a lack of information about glucose. As an example, Figure 2 shows how information about ammonium and glucose abundance (x- and y-axes) interact to produce observed growth rate differences (z-axis). If ammonium sensing is highly accurate, then increasing information about glucose concentrations causes a linear increase in fitness (the plane parallel to the yz-axes, at an accuracy of 24 bits for ammonium availability) exactly as in Figure 1. However, if ammonium sensing is poor (the same plane, but at zero bits of ammonium information) then good glucose sensing yields no growth-rate gain, because it is the poor ammonium-sensing that effectively limits growth. If ammonium-sensing accuracy is intermediate, then an improvement in glucose sensing causes a growth-rate increase (smaller *s*) up to some number of bits. From that point on, additional glucose-information has no effect, because ammonium-sensing has become growth-limiting. Exactly the same considerations hold if the places of ammonium or glucose are interchanged (or for any other two nutrients), which causes the symmetry of the piecewise linear surface in Figure 2.

**Figure 3:**
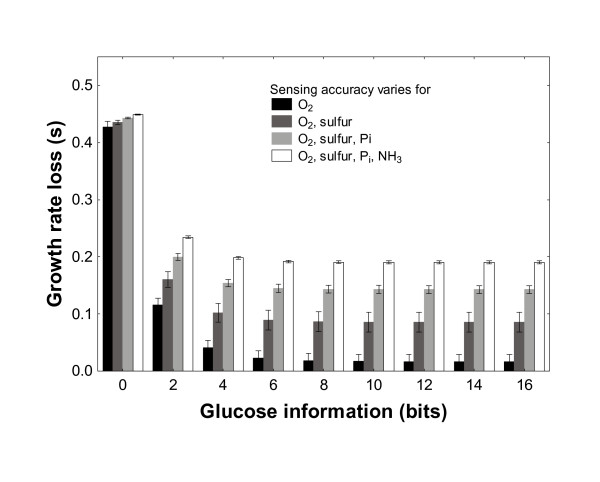
Effect of glucose information (bits) on the selection coefficient *s*, if sensing accuracy for a varying number of other substrates (differently shaded bars) varies uniformly in the interval (0, 16) bits.

Figure 3 shows a different representation of the relationship between the fitness value of information about one growth substrate, and variation in information about another growth substrate. The figure shows how increasing information about glucose concentration (horizontal axis) affects biomass yield and thus fitness (vertical axis), if at the same time information measured about one or more *other *nutrients varies randomly. For example, the black bars correspond to a situation where only one other substrate, oxygen, is measured at accuracies randomly and uniformly distributed between 0 and 16 bits. For increasing glucose information, the growth rate loss becomes smaller, but never as small as when *only *glucose information limits growth (note the logarithmic and linear vertical axes in Figures 1a and 1c, respectively). Even at glucose information of 16 bits, the biomass yield loss by a cell relative to the maximal growth rate is of the order of *s *= 0.02 (2 percent, Figure 3, black bars), several orders of magnitude greater than the *s *≈ 10^-8 ^observed if oxygen sensing is highly accurate. This means that inaccurate sensing of one growth substrate severely limits the value of information about other growth substrates. This limitation becomes more severe as the number of growth substrates for which uncertainty exists increases (black to white bars in Figure 3). Although the data is shown for specific growth substrates, the results are insensitive to the kind of nutrients for which imprecise information is available.

**Figure 4:**
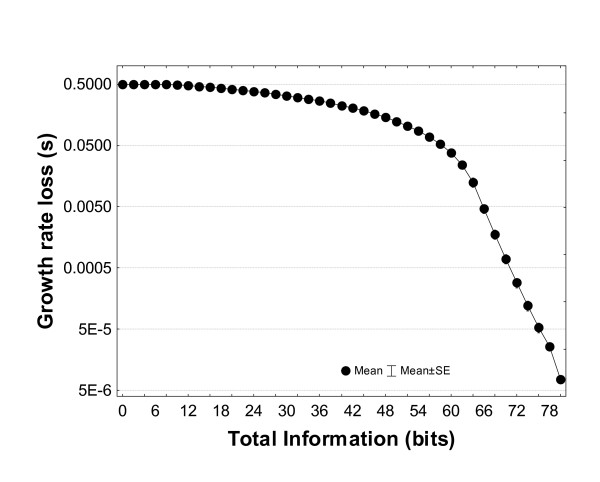
The selection coefficient *s *depends nonlinearly on the total amount of information available for all five substrates. Information about each substrate was varied in the interval (0, 16) bits. All data are for a minimal, aerobic medium with glucose as the sole carbon source and the following five substrates: glucose, O_2_, phosphate, sulfate, and NH_3_.

Figure 4, finally, shows the effect on biomass yield of the total amount of information available, i.e., summed over five key growth substrates in a minimal glucose medium, where information on each growth substrate can vary between 0 and 16 bits. Biomass yield increases (*s *decreases) only slightly as the amount of available information increases, until much information (>60 bits) becomes available, at which point every additional bit has a large effect. This means that the benefits of growth substrate information are limited by the poorest sensing process in a metabolic system. Only if every substrate-sensing process has high accuracy, does increasing information about any one substrate provide large benefits.

## Discussion

The notion that cells process information is not new [33]. However, it is usually expressed qualitatively, without reference to the amounts of information involved and what exactly is being processed. Using metabolic flux balance analysis, I here take a small step towards a quantitative approach to information processing in cells. Specifically, I show that nutrient sensing inaccuracy is translated into reduced cell growth. In the simplest possible case of information limitation in only one nutrient, the relationship between cell growth and information (in bits) is best expressed as -log_2_(*s*) = *I*+1, where *s *is a selection coefficient, the difference between the maximal growth rate with perfect sensing and the actually attained growth rate.

Population genetic considerations show that very small selection coefficients of s < 10^-6 ^are still visible to natural selection in microbes like yeast. Very small inaccuracies (≈ 0.0001 percent measurement error; Figure 1) can thus still lead to growth rate loss with evolutionary consequences. Yeast cells would need to sense nutrients at accuracies greater than 22 bits to ensure optimal growth. Organisms with larger population sizes would need even greater accuracies.

Although nutrient sensing mechanisms are only incompletely understood [23-25], several lines of evidence suggest that the needed accuracies are unlikely to be achievable in practice. First, with an average volume of 9.5 × 10^-14 ^liter for a yeast cell [34], a typical nutrient concentration of 10 mmol l^-1 ^translates into 5.72 × 10^8 ^molecules. Temporal random fluctuations scale as the square root of the number of molecules [35], and will be of the order of 0.004 percent, more than an order of magnitude greater than the necessary accuracy. At physiological nutrient concentrations, the needed sensing accuracy thus is greater than random fluctuations in molecule numbers.

Second, although it is generally unknown how accurately cells can sense molecule concentrations, some benchmarks come from the accuracy with which cells can sense concentration *differences*. Eukaryotic cells, including yeast, can detect concentration differences of 1–10% across the length of a cell [36]. Much smaller *E. coli *cells can detect concentration differences of 0.01% by integrating information over time during chemotactic swimming [37-41]. However, these accuracies are two orders of magnitude or more smaller than the measurement error associated with 22 bit sensing accuracy of absolute nutrient concentrations.

A third line of evidence comes from measurements of gene expression noise. For optimal utilization of a nutrient, several classes of molecules need to be expressed at a minimum level determined by the nutrient concentration. These include the nutrient sensors, the signaling molecules needed to communicate the sensing information to regulators of gene expression, and the nutrient utilization enzymes themselves. Sensing is suboptimal if any one of these classes of molecules is expressed at too low a level. Concentrations of all gene products fluctuate in a cell due to gene expression noise [38-42]. Although highly expressed proteins show low expression noise, even highly expressed yeast proteins may fluctuate in concentration by about 10% around their mean [41]. If concentrations of sensing and utilization molecules need to be fine-tuned for high sensing accuracy, then sufficiently high sensing accuracy is not realistic. In sum, fluctuations of nutrient concentrations, limits to detection of concentration differences, and gene expression noise will conspire to prevent high-accuracy sensing of nutrient concentrations needed for optimal growth.

A number of caveats to this approach are in order. First, I have here emphasized sensing errors that lead to underestimation of substrate availabilities, because only such errors lead to an undercapacity to metabolize nutrients. Overestimation would lead to overcapacity of nutrient utilization systems, which in itself would still lead to maximal growth. However, if overestimation causes a systematic overexpression of signaling or utilization molecules, overestimation could carry an increased cost of gene expression. Even though few genes might be involved, these costs are not necessarily low. For example, in case of the lactose operon of *E. coli*, overexpression of only three lactose utilization genes in the absence of lactose leads to a 5% reduction in growth rate (*s *= 0.05) [26], which is a very large fitness loss compared to the small values visible to natural selection [43]. Hundreds of genes can change expression in response to nutrient availability in yeast [44]. Because many of these genes do not encode metabolic enzymes, it is not straightforward to predict the ensuing change in expression cost from a metabolic model. In addition, available *quantitative *information about expression changes for such genes has very limited accuracy for mRNA, and is generally unavailable for proteins. Thus, although it would be highly desirable to understand both the growth cost of underestiming and overestimating nutrient concentrations [44], a quantitative analysis of such costs must await more complete characterization of sensing pathways and gene expression changes therein.

A second caveat is that selection may act concurrently on multiple attributes of a metabolic system, not only on nutrient sensing. One example comes from glucose limitation experiments in chemostats, where a population's environment is held constant for hundreds of generations. Consider a nutrient whose actual concentration is *N*, and the value of its concentration sensed by a cell (possibly with some inaccuracies) is *S*(*N*). If the import system for this nutrient is far from being saturated, which is likely if the nutrient is at concentrations sufficiently low to be growth limiting, then the cell's uptake rate *U *of this nutrient is likely to be proportional to *S*(*N*), with some proportionality constant *c*, i.e., *U *= *cS*(*N*). In this paper, I focus on the value of improved nutrient sensing *S*(*N*). However, the proportionality constant *c *itself may be subject to selection pressure, thereby increasing the efficiency of uptake for a given *S*(*N*). For example, in yeast populations cultivated during several hundred generations in a chemostat, such increase in uptake efficiency occurs through gene duplication and increased expression of hexose transporter genes [45,46]. Note, however, that the constant chemostat conditions of such experiments are likely to be rare in the wild, where an exponentially growing population rapidly exhausts any limiting nutrient source.

A final caveat is that it may not always be possible to sense the availability of two compounds independently from one another. One example is the sensing of glucose and protons (H^+^), where in yeast the glucose sensor *Snf3p *is known to activate the proton pumping plasma membrane ATPase [47].

Nutrient sensing systems are only as strong as their weakest link: Inaccurate sensing of one nutrient may strongly reduce the fitness benefits of high quality sensing of other nutrients. However, it is easy to see how multiple independent mutations, each in a different nutrient sensing system, may favor incremental improvements in the sensing of all nutrients through natural selection. The reason is that an allele that increases sensing quality for one nutrient will increase fitness whenever that nutrient is limiting, and be driven to fixation during such times. This increases the value of better information for other nutrients, and favors alleles that improve information acquisition for these nutrients, thus increasing the respective mutations in frequency, and so on. At the end point of many such evolutionary cycles stands a cell that achieves the best possible nutrient sensing, given biophysical and population size constraints.

Recent work suggests that the lens of natural selection can see seemingly minute changes in transcriptome and proteome composition, such as single amino acid changes and small changes in the expression of one gene [48]. The observations made here likewise emphasize the importance of natural selection to shape nutrient sensing accuracy. In addition, they suggest the existence of biophysical constraints that may severely limit the outcome of selection on high-accuracy nutrient sensing to biophysically achievable, but suboptimal solutions. This perspective only becomes possible through a system-wide analysis of a metabolic network. An important task of future work would be to *quantify *the constraints natural selection faces in optimizing how cells acquire information.

## Methods

Flux balance analysis [49] uses information about the stoichiometry of all enzymatic reactions known to occur in an organism, which is encapsulated in a stoichiometry matrix **S**. At a metabolic steady-state, the vector of allowable metabolic fluxes **v **describing the rates through each reaction in the network must fulfill the condition **Sv **= 0 so as to not violate mass conservation. For each **v **that is a solution of this equation, c**v **(c being some real constant) is also a solution, such that one can think of **v **as specifying relative *ratios *of fluxes through a metabolic network. Further constraints, such as irreversibility of some reactions, and experimentally measured uptake fluxes of external substrates can reduce the number of allowable fluxes **v **in steady-state. Within the space of allowable fluxes one can then use linear programming to determine the fluxes that maximize or minimize any quantity that can be expressed as linear combinations of individual metabolic fluxes. An especially important such quantity is the biomass growth flux itself.

The following substrate uptake fluxes (mmol substrate/h/g dry weight) were used here (rounded to two significant digits): Glucose: 15.3; O_2_: 2.4; Sulfate: 0.034; inorganic phosphate P_i_: 0.09; ammonium NH_3_: 2.45. Among these values, the glucose uptake flux *v*_G _and oxygen uptake flux *v*_O2 _stem from experimental measurements in an aerated batch culture of *S. cerevisiae *[50]. I constrained these two fluxes and then determined the maximal growth flux *v*_*max *_given these constraints, where the stoichiometry of the growth flux is given in [12]. I then constrained the growth flux to v_max _(while still constraining *v*_G_, *v*_O2 _to the above values), and minimized the sulfate uptake flux for which this growth flux could be observed. The resulting sulfate uptake flux (see value above) is the smallest sulfate uptake flux that can sustain the observed *v*_*max *_with the given glucose uptake flux. I subsequently carried out analogous minimization procedures for the remaining two growth substrates, thus arriving at the values listed above. This combination of values has the advantage that reduction in any one nutrient uptake flux will lead to a reduction in growth rate. In other words, no nutrient is in excess, and accurate nutrient availability estimation is critical to sustain maximal growth. All analyses were carried out with a publicly available yeast metabolic model [12] and with the FBA package "sbrt" (Wright and Wagner, unpublished), using the commercial linear programming package CPLEX (ILOG, Mountain View, CA.). To estimate expected growth rates for an amount of information *I*_*i *_available for a given nutrient *i*, I translated *I*_*i *_into the appropriate number of measurement intervals *n*_*i*_, according to the relation *I*_*i *_= log_2 _*n*_*i *_discussed in the main text. The expected measured nutrient value then calculates as *N*_*i*_(2*n*_*i*_-1)/2*n*_*i*_, where *N*_*i *_is the actual nutrient concentration represented through one of the uptake fluxes listed above. Flux balance analysis was carried out with this expected value to determine the growth rate achievable for the corresponding amount of information.

As stated, nutrient concentrations are here represented through nutrient uptake rates. That is, I implicitly assume that if the concentration of a *limiting *nutrient changes by *x*%, then a cell with access to perfect nutrient sensing would also change its uptake rate of the nutrient by *x*%. However, it must be clarified that nutrient uptake rates are not necessarily proportional to nutrient concentrations, even if cells have perfect information. Specifically, if there is so much of a nutrient that the nutrient uptake transporters are saturated, changes in extracellular nutrient concentrations will have no effect on nutrient uptake. However, this scenario is very different from my focus here, namely an environment where the concentration of individual nutrients limits growth, and where information about such nutrients matters to the cell.

## Authors' contributions

AW designed and executed the research, analyzed the data, and wrote the manuscript.
